# 3-Alkoxy-1-Benzyl-5-Nitroindazole Derivatives Are Potent Antileishmanial Compounds

**DOI:** 10.3390/ijms251910582

**Published:** 2024-10-01

**Authors:** Niurka Mollineda-Diogo, Sergio Sifontes-Rodríguez, María Magdalena Aguirre-García, Alma Reyna Escalona-Montaño, Teresa Espinosa-Buitrago, Ricardo Mondragón-Flores, Mónica Edith Mondragón-Castelán, Alfredo Meneses-Marcel, Ofelia Pérez-Olvera, Daniel Andrés Sánchez-Almaraz, Yunierkis Perez-Castillo, Vicente Arán-Redó

**Affiliations:** 1 Centro de Bioactivos Químicos, Universidad Central “Marta Abreu” de Las Villas, Santa Clara, Villa Clara 250512, Cuba; ameneses@uclv.edu.cu; 2 Instituto de Investigaciones Biomédicas, Universidad Nacional Autónoma de México—Consejo Nacional de Humanidades, Ciencias y Tecnologías (CONAHCYT), Ciudad de México 14080, Mexico; oigresergio@gmail.com; 3 Unidad de Investigación UNAM-INC, Facultad de Medicina, Universidad Nacional Autónoma de México—Instituto Nacional de Cardiología Ignacio Chávez, Ciudad de México 14080, Mexico; maguirre@unam.mx (M.M.A.-G.); almiusqfb23@yahoo.com.mx (A.R.E.-M.); o.perez.olvera@gmail.com (O.P.-O.); chillon_15@yahoo.com.mx (D.A.S.-A.); 4 Facultad de Farmacia, Universidad San Pablo-CEU, CEU Universities, Urbanización Montepríncipe, Boadilla del Monte, 28660 Madrid, Spain; emb.teresa@gmail.com; 5 Departamento de Bioquímica, Centro de Investigaciones y Estudios Avanzados del Instituto Politécnico Nacional (CINVESTAV), Ciudad de México 14080, Mexico; rmflores@cinvestav.mx (R.M.-F.); mmondrag@cinvestav.mx (M.E.M.-C.); 6 Grupo de Bio-Quimioinformática and Facultad de Ingeniería y Ciencias Aplicadas, Universidad de Las Américas, Quito 170125, Ecuador; 7 Instituto de Química Médica del Consejo Superior de Investigaciones Científicas de España, Juan de la Cierva 3, 28006 Madrid, Spain; viaran@jgm.csic.es

**Keywords:** 3-alkoxy-1-benzyl-5-nitroindazole, 2-benzyl-5-nitroindazolin-3-one, leishmaniasis, promastigote, amastigote, antileishmanial activity

## Abstract

Indazoles have previously been identified as molecules with antiprotozoal activity. In this study, we evaluate the in vitro activity of thirteen 3-alkoxy-1-benzyl-5-nitroindazole derivatives (series D) against *L. amazonensis*, *L. infantum,* and *L. mexicana*. In vitro, cytotoxicity against mouse peritoneal macrophages and growth inhibitory activity in promastigotes were evaluated for all compounds, and those showing adequate activity and selectivity were tested against intracellular amastigotes. Transmission and scanning electron microscopy were employed to study the effects of 3-alkoxy-1-benzyl-5-nitroindazole and 2-benzyl-5-nitroindazolin-3-one derivatives on promastigotes of *L. amazonensis*. Compounds NV6 and NV8 were active in the two life stages of the three species, with the latter showing the best indicators of activity and selectivity. 3-alkoxy-1-benzyl-5-nitroindazole derivatives (series D) showed in vitro activity comparable to that of amphotericin B against the promastigote stage of *Leishmania* spp. Two compounds were also found to be active the amastigote stage. Electron microscopy studies confirmed the antileishmanial activity of the indazole derivatives studied and support future research on this family of compounds as antileishmanial agents.

## 1. Introduction

Indazoles are heterocyclic aromatic compounds formed by the fusion of a benzene ring and a pyrazole ring, usually present in two tautomeric forms: 1H-indazole (thermodynamically more stable) and 2H-indazole [1]. Although rare in nature [2], synthetic derivatives of indazole exhibit a broad spectrum of biological activities, including antitumor, antiarrhythmic, anti-inflammatory, and antimicrobial activities [3,4,5,6,7,8]. Therefore, such molecules are of particular interest in view of their potential applications in medical and pharmaceutical research [9]. In addition, the biological activity of indazoles can be modulated depending on the specific core structure and the variety of modifications that can be introduced [10]. Advanced methods have now been designed to synthesize diverse indazole derivatives bearing multiple types of substituents [11]. Consequently, research on indazoles and their biological applications remains active and promising.

The evaluation of indazoles as antiprotozoal agents began with the study of new safe and effective drug candidates for the treatment of Chagas disease, mainly due to their chemical similarity with the reference drugs used in clinical practice [12]. These first reported compounds exploited the combination of an indazole ring and a 5-nitro group due to their previously demonstrated antiprotozoal properties [13,14]. Three series of compounds were designed using different substituents on both the carbon and nitrogen atoms.

**Figure 1:**
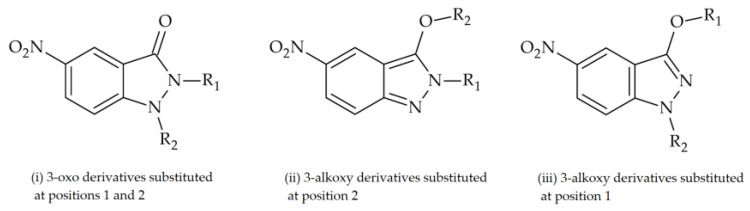
Series of 5-nitroindazolin-3-one structures evaluated against *T. cruzi*.

**Figure 2:**
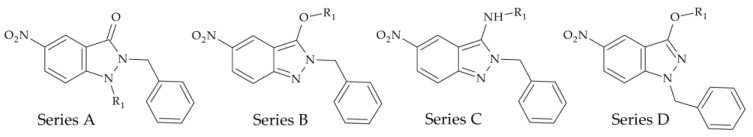
New series of compounds designed for inhibition studies of *T. cruzi*.

In a previous study, three series of 5-nitroindazolin-3-one derivatives were evaluated for bioactivity against *T. cruzi* (Figure 1): (i) 3-oxo derivatives substituted at positions 1 and 2; (ii) 3-alkoxy derivatives substituted at position 2; and (iii) 3-alkoxy derivatives substituted at position 1 [15]. This study made it possible to evaluate the substituents with the highest impact on activity and led to the design of four new series of compounds (Figure 2). 

(A) 20 compounds derived from 1-substituted 2-benzyl-5-nitroindazolin-3-ones [15], 

(B) 5 compounds derived from 3-alkoxy-2-benzyl-2H-indazoles [15], 

(C) 3 compounds derived from 3-(aminoalkyl)-5-nitroindazoles [15], and

(D) 13 compounds derived from 3-alkoxy-1-benzyl-5-nitroindazoles [12].

The compounds in series D were obtained from 1-benzyl-5-nitroindazole-3-ol. During their synthesis, modifications were made at the 3-O position of the 5-nitroindazole scaffold with the aim of improving the activity, solubility, and stability of the previous series (A, B, and C) [16,17]. The introduction of piperidine substituents was justified by their demonstrated activity in the production of radicals capable of inducing oxidative stress in *T. cruzi* [17]. Another aspect considered during the design of series D was to maintain the bioactivity of the previous series while improving their solubility and bioavailability [17]. Consequently, compounds with alkylamine substituents that are expected to be more soluble in water were designed [17].

Considering the antiprotozoal activity shown by these new indazole derivatives, particularly against *T. cruzi* and *T. vaginalis* [16,17,18,19,20], and preliminary evidence of some of these compounds against *L. amazonensis* [17,21], we evaluate in the present work the in vitro activity of 3-alkoxy-1-benzyl-5-nitroindazoles (series D) on *L. amazonensis*, *L. infantum*, and *L. mexicana*. We also report the structural and ultrastructural damage caused by these compounds and 2-benzyl-5-nitroindazolin-3-one derivatives (series A) in *L. amazonensis*.

## 2. Results

**Figure 3:**
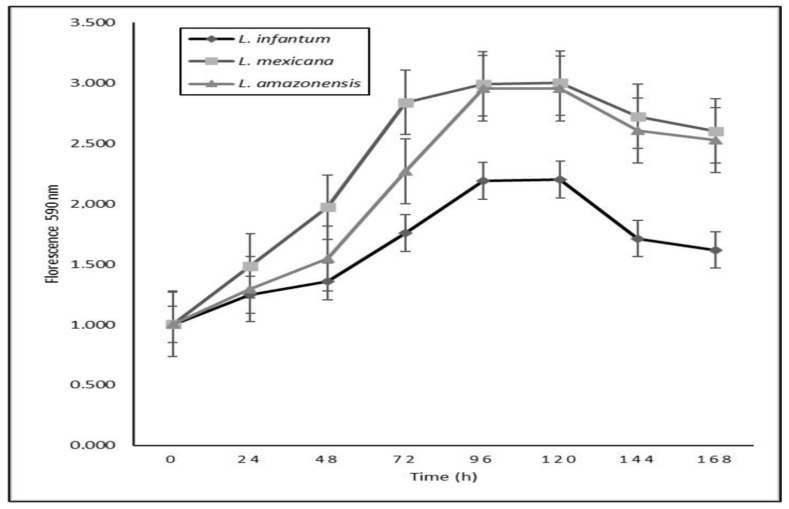
Growth curves of the promastigotes of the three species of *Leishmania*.

The three strains of *Leishmania* showed similar growth patterns (Figure 3). Growth behavior is characterized by a first adaptation or dormancy of less than 24 h, followed by a phase of exponential growth, where the crop multiplies to its maximum potency that lasts approximately three days in the period of 24–96 h after the establishment of the culture. Next, the growth of the culture is unbalanced in a deceleration or stationary phase from 96 to 120 h and a cell death phase beginning after 120 h. Based on these results, an incubation period of 72 h was established to evaluate the in vitro effects of the compounds against the promastigotes of these species.

Out of the thirteen compounds derived from 3-alkoxy-1-benzyl-5-nitroindazole, eight had a mean cytotoxic concentration (CC_50_) > 20 μM, and five had a CC_50_ < 10 μM (Table 1). Compounds whose substituent was a tertiary, secondary, or primary amine bound to the 3-O position by a pentyl group, such as NV14, NV15, and NV17, showed increased cytotoxicity compared to those with tertiary, secondary, or primary amines bound to the 3-O position by an ethyl or propyl group, such as NV4 and NV6. The introduction of bromoalkyl groups at the 3-O position also induced an increase in cytotoxicity in compounds NV11 and NV12.

The in vitro inhibitory activity of the compounds against promastigotes of different species of *Leishmania* (Table 2) was potent, with a high selectivity index. Compounds with a mean inhibitory concentration (IC_50_) ≤ 1 μM and selectivity index (SI) ≥ 10 at the promastigote stage were considered active. Eight compounds against *L. amazonensis* (NV4, NV6, NV7, NV8, NV9, NV10, NV13 and NV16), eight against *L. infantum* (NV4, NV6, NV7, NV8, NV9, NV10, NV13, and NV17), and seven chemicals against *L. mexicana* (NV6, NV7, NV8, NV13, NV14, NV15 and NV16) met these criteria. 

In the intracellular amastigote system, eleven compounds that met the activity criteria defined for promastigotes were studied (Table 2). Only compounds NV11 and NV12 were classified as inactive against promastigotes for all *Leishmania* species. Against intracellular amastigotes, the compounds selected as active were those with values of average effective concentration (EC_50_) ≤ 5 μM and SI ≥ 10: five compounds against *L. amazonensis* (NV6, NV8, NV9, NV10, and NV16), four against *L. infantum* (NV4, NV6, NV8, and NV10), and two against *L. mexicana* (NV6 and NV8). Compounds NV6 and NV8 were active in amastigotes of all three species, with NV8 showing the best indicators of activity and selectivity against the three species simultaneously.

ADMET (Absorption, Distribution, Metabolism, Excretion, and Toxicology) properties were predicted with the ADMETboost web server and OSIRIS Property Explorer. This software was later employed to compute a “drug score” predicting whether a molecule is a good drug candidate. Amphotericin B is the leading compound currently used in the treatment of leishmaniasis; thus, indicators of its in vitro activity constitute a reference standard for the selection of antileishmanial compounds. This justifies its use in this research to establish comparisons with indazole derivatives from a pharmacokinetic point of view. The results of ADMET predictions for the compounds reported here and the reference drug amphotericin B are provided in Table 3.

Overall, the most favorable physicochemical properties for oral bioavailability are observed for compounds NV4, NV6, NV7, NV8, NV9, NV10, and NV13. The rest of the chemicals in the series show unfavorable n-octanol-water partition coefficients (log KOW > 3). However, all the compounds are predicted to have better oral bioavailability than the reference drug AmB. Parameters such as those related to absorption, distribution, metabolism, and excretion are similar among the reported compounds and AmB. In terms of drug score, the most favorable values are obtained for compounds NV4, NV6, NV9, and NV10. None of the active compounds were classified as posing a risk of potential mutagenic, tumorigenic, irritant, or reproductive effects (Table 3). Compounds with bromoalkyl substituents (NV11 and NV12), which were not active and were considered cytotoxic (CC_50_ < 10 μM), are predicted to have a high risk of inducing mutagenic, tumorigenic, irritant, and reproductive effects.

**Figure 4:**
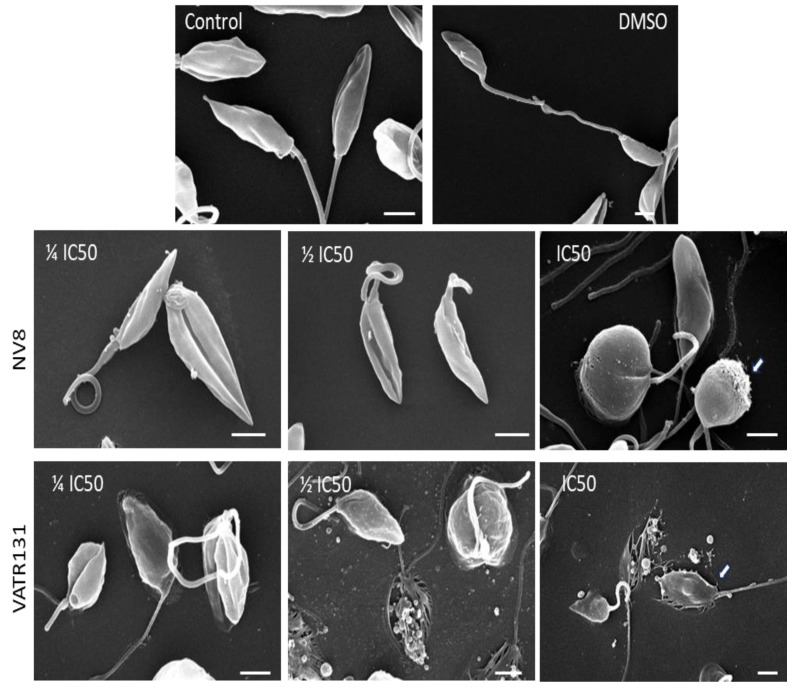
Scanning electron microscopy of NV8 (IC_50_ = 0.007 μM, ½ IC_50_ = 0.0035 μM, ¼ IC_50_ = 0.0017 μM) and VATR 131 (IC_50_ = 2.13 μM, ½ IC_50_ = 1.065 μM, ¼ IC_50_ = 0.53 μM) against promastigotes of *L. amazonensis*. Scale bar = 0.5 µm.

Figure 4 shows the results of scanning electron microscopy at concentrations of the compounds corresponding to IC_50_, ½ IC_50,_ and ¼ IC_50_. Control promastigotes and those treated with 0.1% DMSO showed no differences. In both conditions, the parasites showed the characteristic morphology of elongated bodies with a pointed shape, average length of 5.5–6 µm, and a polar flagellum. The flagellum was 6.5–9 µm long and generally had an extended appearance. Eventually, small vesicles of 0.5 microns in diameter were detected, usually associated with the junction zone between the parasite and the flagellum. When promastigotes were treated with compound NV8, the appearance of the flagellum changed, even at the lowest concentration (¼ IC_50_), and appeared in a coiled form and shortened in length. In addition, the body of the promastigotes shows an obvious deformation that reaches its maximum at a concentration equal to IC_50_. At this concentration, the bodies were completely swollen and spheroidal in appearance, although their shortened flagella were preserved. In some cases, the surface of the parasites shows damage to the plasma membrane in the form of perforations (white arrow).

When the promastigotes were treated with compound VATR131 at different concentrations (Figure 4), an alteration in the morphology of the parasites was observed from the lowest concentration of the compound. The shortening of the flagella is evident, with irregular undulations and a clear alteration in the morphology of the body of the promastigotes. Many of the parasites appear to be strongly attached to the surface of the substrate, with evident crushing of the body (white arrow).

As the concentration of the compound increases, destruction of the parasite is observed, leaving remnants of subcellular organelles attached to the surface of the substrate. All parasites were deformed at a concentration of the compound equal to its IC_50_ and crushed on the substrate, suggesting instability of the membrane and, most likely, of the cytoskeleton that supports their shape.

**Figure 5:**
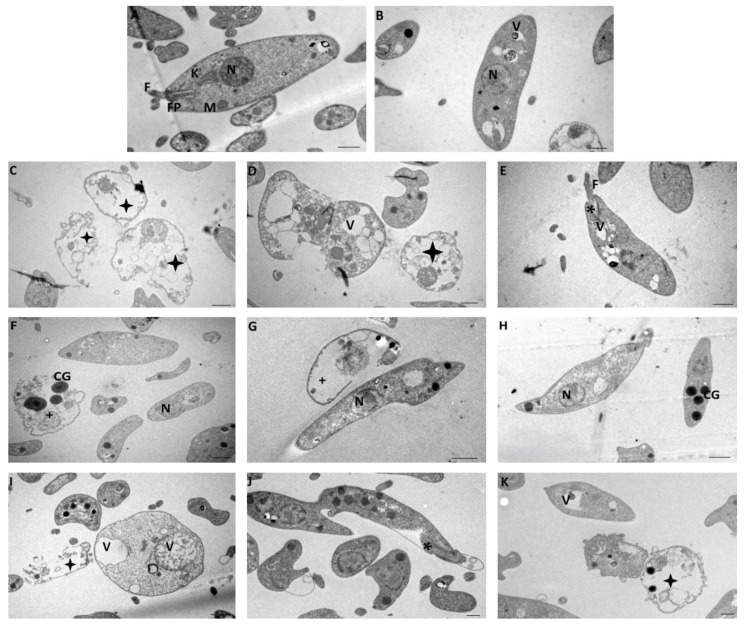
Transmission electron microscopy of *L. amazonensis* promastigotes after 72 h of incubation: Control Group (**A**), DMSO 0.1% (**B**), IC_50_ = 0.007 μM (**C**–**E**), ½ IC_50_ = 0.0035 μM (**F**–**H**), and ¼ IC_50_ = 0.0017 μM (**I**–**K**) of NV8 compound. Scale bar = 0.5 µm. Flagellum (F), Flagellar Pocket (FP), Kinetoplast (K), Nucleus (N), Mitochondria (M), Cytoplasmic Vacuoles (V), Dead Parasites (black star), Deformed Flagellar Pocket (*), Dead Parasites with extruded cytoplasm (+) and Cytosolic Granules (CG).

Figure 5 shows images of transmission electron microscopy to study the effect of different concentrations of NV8 on *L. amazonensis* promastigotes. Image A corresponds to the untreated Control Group, where the parasites show their characteristic morphology, retaining their flagellum (F), flagellar pocket (FP), kinetoplast (K), nucleus (N), and mitochondria (M). Image B shows the group treated with DMSO (0.1%), where the parasites maintain their characteristic shape, but cytoplasmic vacuoles (V) contain degraded material. These vacuoles are similar to autophagic vacuoles, but are not present in all parasites. The damage caused by ¼ IC_50_ can be seen in images C-E, which include many dead (black star) and swollen parasites with large, dense, deformed granules, while some parasites retained their flagellum despite presenting deformation of the flagellar pocket (*). The group treated with ½ IC_50,_ corresponding to images F-H, presents dead parasites with extruded cytoplasm (+) and swollen and apparently normal parasites. There are deformed parasites with large cytosolic granules (CG), and few parasites have retained their flagellum. Finally, in images I-K showing the group treated with a concentration of the compound equal to its IC_50_, most of the parasites are atypical; many are swollen or deformed and present large cytosolic vacuoles (V). The presence of dense granules of 300 to 400 nm in diameter is also detected. There are dead parasites with extruded cytoplasmic content, and several parasites have membrane separation and loss of flagella.

**Figure 6:**
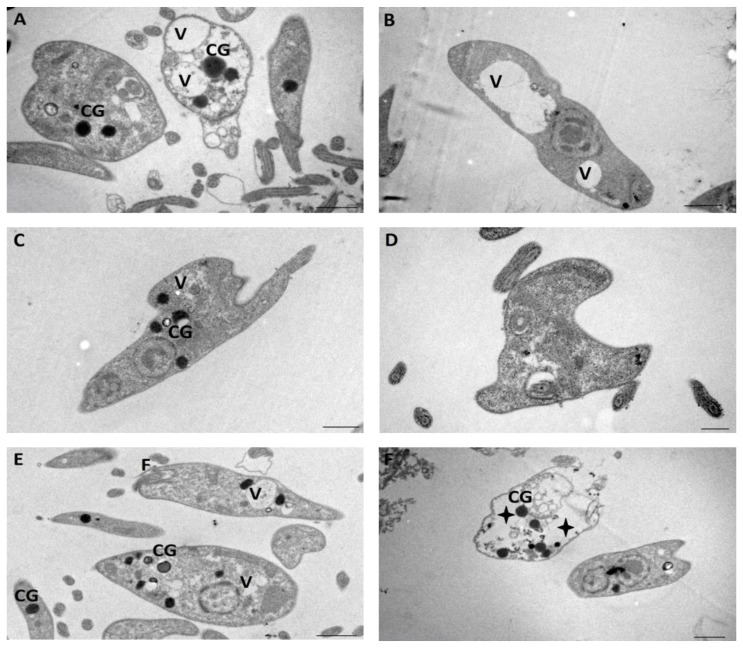
Transmission electron microscopy of *L. amazonensis* promastigotes after 72 h of incubation with ¼ IC_50_ = 0.53 μM (**A**,**B**), ½ IC_50_ = 1.065 μM (**C**,**D**) and IC_50_ = 2.13 μM (**E**,**F**) of compound VATR 131. Scale bar = 1 µm.

Images of the cultures treated with different concentrations of compound VATR 131 are shown in Figure 6. Cultures treated with ¼ IC_50_ are presented in A and B, while C and D correspond to treatment with ½ IC_50_, and E and F show the effect of treatment with a concentration equal to IC_50_. The effects of the treatments on the culture of promastigotes are very similar to those observed for NV8 at concentrations equivalent to ¼ IC_50_ and ½ IC_50_. However, the degree of damage severity in the crop treated with NV8 at a concentration equal to its IC_50_ was much higher than that in the culture treated with the equivalent concentration of VATR131.

## 3. Discussion

The compounds reported here are characterized by their low cytotoxicity against primary cultures of mouse macrophages, although their cytotoxic profile is less favorable against Vero cells [19]. Compared to 2-benzyl-5-nitroindazolin-3-one derivatives (Series A), the compounds studied here showed lower cytotoxicity in vitro than in primary cultures [21]. Of all compounds, eight (61.5%) met the international criteria defined for selecting candidates with antileishmanial activity against the promastigotes stage (IC_50_ < 1 µM and SI ≥ 10) against *L. amazonensis* and *L. infantum* and seven (53.8%) against *L. mexicana* [22]. Four compounds (30.7%) with in vitro activity against the three *Leishmania* species studied (NV6, NV7, NV8, and NV13) stand out within the series. These results support the antileishmanial potential of the derivatives studied, since compounds designed by other authors with the same pharmacophore group have not shown potent activity. In this sense, derivatives of 5-nitroindazole showed IC_50_ values higher than the indazoles reported here against *L. infantum* and *L. braziliensis* [23], with values between 11 and 52 μM. Our results show that compounds NV7 and NV8 have IC_50_ values comparable to that of AmB at the promastigote stage. These compounds are also more active than other drugs used in the clinic, such as pentamidine (IC_50_ = 30 μM) [24] and miltefosine (IC_50_ = 50.7 μM) [25]. In terms of the selectivity index, the NV8 compound is of particular interest as it shows values comparable to AmB in both life stages of the three *Leishmania* species studied.

Initial studies in promastigotes were the first to rule out inactive compounds and advance the evaluation of the active ones against the intracellular amastigote system. The D-series also turned out to be much more active compared to the series A in amastigotes [21]. Five compounds showed EC_50_ ≤ 5 μM and IS > 10 against *L. amazonensis* (NV6, NV8, NV9, NV10, and NV16), four against *L. infantum* (NV4, NV6, NV8, and NV10), and two against *L. mexicana* (NV6 and NV8). Two of the most active compounds at the promastigote stage of the three species (NV6 and NV8) were also active against amastigotes. NV8 was found to be the most active compound in the promastigote stage, while in the amastigote phase of *L amazonensis* NV6 displayed better results. This could be caused by the difference in the substituents present in the two compounds. In the case of NV8, bulky piperidine and saturated heterocyclic amine groups are present, while NV6 contains a tertiary amine that potentially favors rotation and internalization in the macrophage membrane. Compound NV8 showed the best indicators of activity and selectivity against *L. infantum* (EC_50_ = 1.26 μM and SI = 18) and *L. mexicana* (EC_50_ = 1.00 μM and SI = 23).

The potency of these compounds in amastigotes is similar to or better than that of different synthetic products that are currently being studied as new antileishmanial alternatives. For example, against amastigotes of *L. amazonensis*, quinoline [26] showed EC_50_ = 0.54 μM, aminoquinoline derivatives [27] have shown EC_50_ values of 8.1 μM and 15.6 μM, ravuconazole [28] have EC_50_ equals to 5.11 μM, and oxoquinoline derivatives [29] have been reported with EC_50_ values between 0.7–2 μM. Although none of the compounds showed EC_50_ values lower than that of the reference drug AmB in intracellular amastigotes of *L. infantum*, four compounds were active and presented better SI. The latter is an important aspect since the cytotoxicity of AmB is one of its main drawbacks for clinical use [30]. 

Although NV8 and AmB display equivalent inhibitory potency against promastigotes, the latter is much more potent at the amastigote stage. These differences in amastigotes could be caused by the lower toxicity profile of NV8. Previous studies have confirmed that the sensitivity of cells to drugs such as AmB varies with cell type [31]. U-937 cells, a cell line isolated from the lymphoblast lung of humans, for example, showed lower sensitivity to AmB compared to other cell lines, such as J774A.1 (murine cell line), TPH-1 (human monocytic cell line), and Vero cells [31]. In this sense, future studies should aim to establish which cell line is most suitable to replace primary cultures in studies evaluating chemical compounds against *Leishmania*. 

In terms of the structure-activity relationships of the indazole derivatives studied, better selectivity values against the promastigote stage are obtained for compounds combining tertiary amines at the 3-O position of the indazole ring and small alkyl groups (NV4 and NV6). The latter also holds true for chemicals bearing cyclic tertiary amines (NV7 and NV8). In the amastigote stage, compounds with tertiary amines (NV6) and cyclic tertiary amines (NV8) at the 3-O position that include small alkyl groups achieve the best selectivity values. Since the best selectivity indices are observed for structures having small alkyl groups, it can be speculated that the length of the (CH_2_)_n_ chain is decisive for the activity of this series against *Leishmania*. Additional data and studies beyond the scope of the current research, including the synthesis and evaluation of new derivatives, will be necessary to establish a complete and accurate structure-activity relationship for this series of compounds.

The evaluation of indazole derivatives against *Leishmania* was inspired by previous studies on *T. cruzi* [16], considering the relationship between *Trypanosoma* and *Leishmania*. In these, we found cross-activity in *L. amazonensis* and *T. cruzi* for five compounds in series A [21]. The derivatives of 3-alkoxy-1-benzyl-5-nitroindazol (D-series) have been previously evaluated against *T. vaginalis* [20], and as a result of our research, we found cross-activity against the promastigote stage in five of the compounds investigated (NV9, NV10, NV13, NV14, and NV15) and cross-activity against the amastigote stage in two (NV9 and NV10). The D-series was characterized by compounds that were very active against *Leishmania* spp., some of them with inhibitory activity comparable to the reference drug and a high selectivity index; therefore, we consider their evaluation against *T. cruzi* very timely due to the genetic proximity between both trypanosomatids. The discovery of a compound with cross-biological activity between leishmaniasis and trypanosomiasis would be very useful, given the co-infection between *Leishmania* and *Trypanosoma cruzi* that occurs in many areas where both diseases are endemic.

On the other hand, ADMET predictions show many favorable properties for the tested compounds. These predictions indicate that the selected compounds are active without mutagenic risk despite the presence of the nitro group, which has limited the discovery of drugs containing nitro groups [32]. These results will be considered in future optimization experiments, where special attention must be paid to unfavorable parameters, such as aqueous solubility and interaction with cytochromes.

The EM study of compounds VATR 131 and NV8 confirmed the leishmanicidal activity of the indazole derivatives. Both compounds alter the morphology and ultrastructure of the parasite. The involvement of these altered structures in the life cycle of the parasite can provide important information for determining the possible targets of these compounds in future experiments [33]. Although the mechanism of action of indazole derivatives on *Leishmania* is still to be defined, the findings of electron microscopy allow us to speculate on a mechanism related to the destruction of parasites due to the alteration of osmotic regulation without affecting the cytoskeleton. The 5-nitro substituent in the indazole ring seems to be essential for the biological activity of these compounds, as it has been suggested that their mechanism of action against trypanosomatids [17] involves the generation of reactive oxygen species (ROS) that can, directly and indirectly, affect the structure and function of macromolecules [14]. The electron microscopy results obtained in our research could be a consequence of such a mechanism of action. However, the presence of the 5-nitro group alone is not enough for the antiprotozoal activity due to solubility and stability issues. These drawbacks can be addressed by incorporating different functional groups into the indazole ring, resulting in compounds with improved biological activity and pharmacological properties. Considering these results, future studies should focus on clarifying the antileishmanial mechanism of action of indazole derivatives. 

## 4. Materials and Methods

### 4.1. Chemistry

Thirteen compounds derived from 3-alkoxy-1-benzyl-5-nitroindazole belonging to series D were evaluated (Table 1). The compounds were synthesized by the Institute of Medicinal Chemistry of the Spanish National Research Council, Madrid. The compounds were prepared from 1-benzyl-5-nitroindazole-3-ol. The details of their synthesis and characterization can be found in [20]. All products had purities greater than 95%. For the in vitro assays, the compounds were dissolved in dimethyl sulfoxide (DMSO, Sigma-Aldrich, St. Louis, MO, USA) at initial concentrations of 1, 10, and 50 mg/mL [15,16,17]. AmB sodium deoxycholate was used as a positive control in all experiments (Julio Trigo López Pharmaceutical Laboratory Company, Havana, Cuba). AmB was dissolved in sterile distilled water at a concentration of 2 mg/mL and stored at 4 °C until its time of use in each experiment.

### 4.2. Parasites

The strains used in this research were MHOM/BR/77/LTB0016 of *L. amazonensis* from the Pedro Kouri Institute of Tropical Medicine Cuba strains collection, MHOM/FR/78/LEM75 of *L. infantum* from the Department of Parasitology, Faculty of Pharmacy of the Complutense University of Madrid Spain, and MNYC/BZ/62/M379 of *L. mexicana* from the Laboratory of Molecular Immunobiochemistry and Heart Diseases UNAM-INC Mexico Research Unit. The parasites were obtained from the skin lesions of previously infected BALB/c mice and maintained at 26 °C in Schneider medium (SIGMA, St. Louis, MO, USA) and supplemented with antibiotics (penicillin sodium 200 IU/mL and streptomycin 200 μg/mL SIGMA, St. Louis, MO, USA) and 10% fetal bovine serum (FBS, SIGMA, St. Louis, MO, USA), and inactivated with heat (56 °C, 30 min). The promastigotes, once isolated, were kept in the exponential multiplication phase by successive subcultures every 3 or 4 days until the time of use. For the experiments, the number of parasites did not exceed 10 subcultures in vitro.

### 4.3. Growth Curves

Promastigote cultures of *L. infantum*, *L. amazonensis*, and *L. mexicana* were adjusted to 10^6^ promastigotes/mL. Seven plates with 96 wells were seeded at 200 μL/well and incubated at 26 °C. A total of 32 replicates/plate were prepared for each species. Every 24 h, 20 μL/well of a 3 mM resazurin solution prepared in sterile PBS (Phosphate Buffer Solution) was added. The reading was performed at 540 nm excitation and 590 nm emission wavelengths after 8 h of incubation in a 96-well plate reader (SUMA^®^, Habana, Cuba). The mean daily fluorescence was obtained for each species and plotted as a function of time (hours).

### 4.4. Peritoneal Macrophage Extraction

To obtain peritoneal macrophages, the mice were sacrificed by cervical dislocation, and the resident cells in the peritoneum were extracted by washing with RPMI (Roswell Park Memorial Institute, Buffalo, NY, USA) medium and cold (4 °C) supplemented with 10% FBS and antibiotics (sodium penicillin 200 IU/mL and streptomycin 200 μg/mL). Cell concentration was calculated using Neubauer chamber counts in an optical microscope (Motic, Xiamen, China) with a 40× objective and 10× eyepieces (400× magnification).

### 4.5. In Vitro Assays

Cytotoxicity in mouse peritoneal macrophages: Peritoneal macrophages were adjusted to 10^5^ macrophages/mL, and 200 μL was seeded per well in a 96-well plate and incubated at 33 °C and 5% CO_2_ for 2 h. Subsequently, the non-attached cells were discarded, and the monolayer was washed with PBS. The compounds were tested in serial dilutions of 1/2.5 (650–1.0 μM). The treated cultures were incubated for 48 h at 33 ⁰C in a humidified atmosphere containing 5% CO_2_.

Susceptibility assay in promastigotes: Promastigotes culture in the exponential growth phase were seeded in 96-well plates (10^6^ parasites/mL) at a final volume of 200 μL/well. Compounds were tested in serial 1/2.5 dilutions (130–0.2 μM). The treated cultures were incubated for 72 h at 26 °C.

Intracellular amastigote assay: Macrophages were seeded in 96-well plates at an initial density of 10^5^ cells/mL in a final volume of 200 µL per well. After 2 h of incubation at 33 °C and 5% CO_2_, the cell monolayer was infected with a culture of promastigotes in the stationary phase of growth at a ratio of 10:1 (promastigotes: macrophages) and incubated under the same conditions for 4 h. The compounds were tested in serial dilutions of 1/2.5 (15–0.02 μM). The treated cultures were then incubated for 48 h under similar conditions. After incubation, the RPMI medium was removed and replaced with Schneider’s medium. Cultures were incubated for 72 h at 26 °C to allow the surviving amastigotes to transform into promastigotes and replicate [34].

Positive (AmB) and negative (DMSO 0.1%) control wells were used for the in vitro studies. Every concentration of the test compounds was assessed in quadruplicate.

Cell viability was assessed using the resazurin transformation method [35]. After the incubation period, 20 μL of 3 mM resazurin solution prepared in sterile PBS was added to each well. Reading was performed at 540 nm excitation and 590 nm emission wavelengths after 8 h of incubation in a 96-well plate reader (SUMA^®^, Habana, Cuba). The mean fluorescence intensity was calculated for each concentration. From the mean values of fluorescence and the corresponding product concentrations, a non-linear fit to the Emax sigmoid model [36] was performed, and the mean inhibitory concentrations (IC_50_), mean effective concentrations (EC_50_), and the mean cytotoxic concentration (CC_50_) were estimated. The selectivity index was calculated as SI_p_ = CC_50_/IC_50_ and SI_a_ = CC_50_/EC_50_ for the promastigote and amastigote stages, respectively.

Only compounds that met the selection criteria of IC_50_ < 1 μM and SI ≥ 10 [22] against promastigotes were advanced to studies in the amastigote stage.

### 4.6. Prediction of ADMET Properties

Pharmacokinetic parameters such as absorption, distribution, metabolism, and excretion were predicted using the ADMETboost web server [37], and toxicological properties such as mutagenic, tumorigenic, irritant potential, and harmful effects on reproduction were predicted using the OSIRIS Property Explorer [38]. This latter program also allows the calculation of a “drug score” that combines toxicological and physicochemical properties to define a score predicting the suitability of candidate molecules to become a drug. A comparison was made with the AmB data from all these parameters. 

### 4.7. Electron Microscopy

The concentrations of compounds selected for the study of structural and ultrastructural damage were those corresponding to IC_50_, ½ IC_50,_ and ¼ IC_50_ for compound NV8 (reported in this study) and the previously reported compound VATR 131 [21]. The procedure for the treatment of cultures was the same as that described above for the in vitro tests on promastigotes of *L. amazonensis*. Two negative controls were used: a DMSO-treated culture (0.1%) and an untreated culture.

#### 4.7.1. Scanning Electron Microscopy

At the end of the exposure to the compounds, the parasites were washed once with PBS and immediately fixed with 2.5% glutaraldehyde in PBS for 1 h at room temperature. After a thorough wash with PBS, the parasites were fixed with 1% OsO_4_ in PBS at 4 °C for 1 h and then washed with PBS. The parasites were dehydrated in increasing concentrations of ethanol. Drying was carried out at the critical point of CO_2_ in a Samdry 780 apparatus (USA). Next, they were evaporated with gold in a Denton Vacuum Desk (USA). Finally, the samples were observed and micrographed using a JSM 6510LB-JEOL microscope at 30 keV.

#### 4.7.2. Transmission Electron Microscopy

Promastigotes were washed with PBS and fixed with 2.5% glutaraldehyde solution for 1 h at 4 °C. They were then fixed with a solution of 1% OsO_4_ in the same buffer at 4 °C and in darkness for 1 h. Promastigotes were washed with PBS at RT and dehydrated in an ethanolic solution in increasing order by up to 100%. They were then included in the epoxy resin using specific molds for polymerization and maintained at 60 °C for a minimum period of 72 h. The blocks were cut with an ultramicrotome (Reichert Jung, Austria) into thin sections. The slices were stained with uranyl acetate and lead citrate and subsequently micrographed using a JEM 1400 transmission electron microscope (JEOL, Ltd., Tokyo, Japan).

## 5. Conclusions

Compounds derived from 3-alkoxy-1-benzyl-5-nitroindazoles (series D) showed in vitro activity against the promastigote stage of three *Leishmania* species comparable to that of amphotericin B. Two of these compounds were also active against the amastigote stage of the three species. Electron microscopy studies confirmed the antileishmanial activity of NV8 (series D) and VATR 131 (series A) derivatives, which caused significant concentration-dependent structural damage. ADMET predictions showed many favorable properties of the compounds selected as active agents, and these results will be taken into account in future optimization experiments. This supports future research into the mechanism of action of this novel family of compounds and potential antileishmanial agents, making significant progress toward the discovery of new, safer, and more effective drugs.

## Figures and Tables

**Table 1:** Chemical structure and in vitro cytotoxicity of the 3-alkoxy-1-benzyl-5-nitroindazole derivatives and amphotericin B.

Base Structure	Product	Substituent R_1_	CC_50_ ± SD (µM)
** 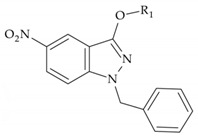 **	NV4	(CH_2_)_2_-N(CH_3_)_2_	67.1 ± 8.9
	NV6	(CH_2_)_3_-N(CH_3_)_2_	61 ± 6.9
	NV7	(CH_2_)_2_-Piperidine	24.4 ± 6.7
	NV8	(CH_2_)_3_-Piperidine	23.2 ± 5.0
	NV9	(CH_2_)_3_-NH_2_	47.9 ± 10.4
	NV10	(CH_2_)_2_-NH_2_	46.7 ± 7.7
	NV11	(CH_2_)_2_-Br	4.5 ± 1.0
	NV12	(CH_2_)_5_-Br	4.1 ± 0.6
	NV13	(CH_2_)_2_-NHCH_3_	61.4 ± 9.4
	NV14	(CH_2_)_5_-N(CH_3_)_2_	2.5 ± 0.7
	NV15	(CH_2_)_5_-NHCH_3_	5.6 ± 2.5
	NV16	(CH_2_)_5_-Piperidine	21.1 ± 7.1
	NV17	(CH_2_)_5_-NH_2_	7.74 ± 3.0
	AmB		5.8 ± 0.5

The values are expressed as the mean CC_50_± SD. CC_50_: Mean cytotoxic concentration is the concentration that causes lysis of 50% of BALB/c mouse peritoneal macrophages. This is the result of averaging at least three experiments. AmB: Amphotericin B.

**Table 2:** Antileishmanial activity of 3-alkoxy-1-benzyl-5-nitroindazole derivatives on promastigotes and amastigotes of *L. amazonensis, L. infantum*, and *L. mexicana*.

**PROMASTIGOTES**						
**Species**	***L. amazonensis***		***L. infantum***		***L. mexicana***	
**Products**	**IC_50_ ± SD (µM)**	**SIp**	**IC_50_ ± SD (µM)**	**SIp**	**IC_50_ ± SD (µM)**	**SIp**
NV4	**0.4 ± 0.1**	**127**	**0.52 ± 0.05**	**127**	2.3 ± 0.4	32
NV6	**0.43 ± 0.007**	**61**	**0.69 ± 0.063**	**45**	**0.28 ± 0.04**	**121**
NV7	**0.04 ± 0.01**	**653**	**0.33 ± 0.021**	**74**	**0.2 ± 0.08**	**14**
NV8	**0.007 ± 0.001**	**3467**	**0.22 ± 0.003**	**104**	**0.11 ± 0.04**	**208**
NV9	**0.81 ± 0.17**	**58**	**0.59 ± 0.16**	**87**	2.8 ± 0003	17
NV10	**0.99 ± 0.14**	**54**	**0.93 ± 0.27**	**51**	4.5 ± 0.5	12
NV11	20.7 ± 1.38	2	20.4 ± 1.3	0.2	9.38 ± 0.13	0.5
NV12	2.88 ± 1.47	2	32.96 ± 0.25	0.1	2.8 ± 1.1	1
NV13	**0.82 ± 0.27**	**74**	**0.29 ± 0.1**	**223**	**0.3 ± 0.04**	**223**
NV14	0.5 ± 0.22	6	0.93 ± 0.18	3	**0.05 ± 0.01**	**55**
NV15	1.88 ± 1.34	4	1.27 ± 0.06	4.4	**0.1 ± 0.01**	**80**
NV16	**0.3 ± 0.22**	**49**	1.41 ± 0.03	15	**0.5 ± 0.1**	**51**
NV17	1.01 ± 0.39	8	**0.20 ± 0.003**	**38**	2.9 ± 0.1	3
AmB	**0.03 ± 0.01**	**193**	**0.28 ± 0.01**	**21**	**0.046 ± 0.012**	**126**
**AMASTIGOTES**						
**Species**	***L. amazonensis***		***L. infantum***		***L. mexicana***	
**Products**	**EC_50_ ± SD (µM)**	**SIa**	**EC_50_ ± SD (µM)**	**SIa**	**EC_50_ ± SD (µM)**	**SIa**
NV4	12.3 ± 0.7	5	**3.42 ± 1.8**	**20**	NE	
NV6	**0.43 ± 0.049**	**71**	**3.79 ± 1.46**	**16**	**2.2 ± 0.05**	**14**
NV7	5.61 ± 0.69	5	12.8 ± 0.13	2	3.1 ± 1.05	8
NV8	**1.00 ± 0.48**	**23**	**1.26 ± 0.5**	**18**	**1.0 ± 0.1**	**23**
NV9	**1.13 ± 0.98**	**42**	9.71 ± 0.4	5	NE	
NV10	**4.35 ± 0.9**	**11**	**2.4 ± 0.34**	**194**	NE	
NV11	NE		NE		NE	
NV12	NE		NE		NE	
NV13	5.66 ± 0.53	11	16.2± 0.54	4	11.6 ± 0.7	5
NV14	NE		NE		8.2 ± 0.2	0.3
NV15	NE		NE		1.53 ± 0.13	4
NV16	**0.17 ± 0.042**	**129**	NE		4.29 ± 0.48	5
NV17	NE		11.8 ± 1.04	1	NE	
AmB	**0.034 ± 0.006**	**170**	**0.6 ± 0.21**	**10**	**0.036 ± 0.008**	**725**

The values are expressed as the mean ± SD. IC_50_: mean inhibitory concentration; this is the concentration that reduces the proliferation of promastigotes by 50%. This is the result of at least three experiments. SIp: Selectivity index for promastigotes, calculated as SI = CC_50_/IC_50_. Products selected as active (IC_50_ ≤ 1 μM) or selective (SIp ≥ 10) are highlighted in bold. EC_50_: Average effective concentration: this is the average effective concentration against intracellular amastigotes. It is the result of at least three experiments. SIa: Selectivity index for amastigotes, calculated as SIa = CC_50_/EC_50_. Products selected as active (EC_50_ ≤ 5 μM) and selective (SIa ≥ 10) are highlighted in bold. SD: Standard deviation. NE: No Evaluated. AmB: Amphotericin B.

**Table 3:** ADMET predictions for anti-leishmanial compounds and the reference drug Amphotericin B (AmB).

**Molecule Property**	**Unit**	**NV4**	**NV6**	**NV8**	**NV9**	**NV10**	**NV16**	**AmB**
Molecular Weight	kg/mol	340.15	354.17	394.2	326.14	312.12	422.23	901.56
Number of Heteroatoms		7	7	7	7	7	7	14
Number of Rotatable Bonds		7	8	8	7	6	10	3
Number of Rings		3	3	4	3	3	4	3
Number of HA		6	6	6	6	6	6	14
Number of HD		0	0	0	1	1	0	9
log KOW	log-ratio	2.93	3.32	4.25	2.72	2.33	5.03	4.51
**Absorption**	**Unit**	**NV4**	**NV6**	**NV8**	**NV9**	**NV10**	**NV16**	**AmB**
Caco-2 Permeability	log(cm/s)	−5.05	−5.04	−5.09	−5.35	−5.36	−5.2	−5.45
HIA	%	75.42	73.4	75.73	75.39	76.06	75.7	66.16
Pgp Inhibition	%	46.26	50.9	52.58	48.08	46.75	51.01	41.51
log D7.4	log-ratio	1.99	2	1.92	1.68	1.74	1.91	1.65
Aqueous Solubility	log(mol/L)	−4.25	−4.4	−4.41	−4.1	−4.03	−4.41	−4.16
Oral Bioavailability	%	50.3	48.42	41.73	49.73	50.45	35.75	38.02
**Distribution**	**Unit**	**NV4**	**NV6**	**NV8**	**NV9**	**NV10**	**NV16**	**AmB**
BBB	%	39.3	40.39	39.87	38.68	38.44	38.8	26.41
PPBR	%	49.51	50.1	50.93	49.34	48.56	50.92	41.18
VDss	L/kg	3.64	3.64	4.15	3.33	3.36	4.14	3.85
**Metabolism**	**Unit**	**NV4**	**NV6**	**NV8**	**NV9**	**NV10**	**NV16**	**AmB**
CYP2C9 Inhibition	%	78.25	80	67.88	78.18	70.38	74.12	50.16
CYP2D6 Inhibition	%	95.53	97.23	97.64	89.45	91.47	96.98	96.33
CYP3A4 Inhibition	%	31.87	33.97	29.14	33.95	32.39	31.28	30.95
CYP2C9 Substrate	%	31.29	32.76	30.06	29.4	29.32	30.68	31.7
CYP2D6 Substrate	%	59.39	64.41	53.41	60.29	62.1	52.28	57.32
CYP3A4 Substrate	%	38.43	39.3	36.13	35.46	35.79	36.86	33.09
**Excretion**	**Unit**	**NV4**	**NV6**	**NV8**	**NV9**	**NV10**	**NV16**	**AmB**
Half Life	h	78.88	92.59	79.39	80.43	81.42	79.75	118.71
CL-Hepa	uL∙min^−1^(10^6^ cells)^−1^	44.17	48.83	45.61	57.02	52.74	44.73	41.66
CL-Micro	mL∙min^−1^ g^−1^	42.42	43.52	39.64	43.65	41.2	41.8	46.05
**Toxicity**	**Unit**	**NV4**	**NV6**	**NV8**	**NV9**	**NV10**	**NV16**	**AmB**
hERG Blockers	%	42.2	44.89	46.81	43.1	42.02	47.91	42.12
DILI	%	52.83	51.13	47.9	53.34	53.44	49.41	52.26
LD50	−log(mol/kg)	2.35	2.28	2.21	2.24	2.37	2.11	2.75
**OSIRIS Property Explorer**	**Unit**	**NV4**	**NV6**	**NV8**	**NV9**	**NV10**	**NV16**	**AmB**
Mutagenic		No	No	No	No	No	No	No
Tumorigenic		No	No	No	No	No	No	No
Irritant		No	No	No	No	No	No	No
Reproductive effects		No	No	No	No	No	No	No
Drug score		0.68	0.56	0.37	0.42	0.43	0.31	0.09

BBB, Blood-brain barrier permeation; CL-Hepa, Clearance hepatocyte; CL-Micro, Clearance microsome; DILI, Probability to produce drug-induced liver injury (DILI); hERG Blockers, Probability of being hERG (Human ether-a-go-go-related gene) blocker; HIA, Human intestinal absorption; LD50, Acute toxicity; log D7.4, Lipophilicity; log KOW, Log n-octanol-water partition coefficient; M, Medium; Number of HA, Number of hydrogen bond acceptors; Number of HD, Number of hydrogen bond donors; Pgp Inhibition, P-glycoprotein inhibition; PPBR, Plasma protein binding rate; VDss, Volume of distribution at steady state.

## Data Availability

The original contributions presented in the study are included in the article, further inquiries can be directed to the corresponding author/s.
